# Evidence that ubiquitylated H2B corrals hDot1L on the nucleosomal surface to induce H3K79 methylation

**DOI:** 10.1038/ncomms10589

**Published:** 2016-02-02

**Authors:** Linjiao Zhou, Matthew T. Holt, Nami Ohashi, Aishan Zhao, Manuel M. Müller, Boyuan Wang, Tom W. Muir

**Affiliations:** 1 Department of Chemistry, Princeton University, Princeton, 08544 New Jersey, USA; 2 Laboratory of Synthetic Protein Chemistry, The Rockefeller University, New York, 10065 New York, USA

## Abstract

Ubiquitylation of histone H2B at lysine 120 (H2B-Ub), a post-translational modification first discovered in 1980, plays a critical role in diverse nuclear processes including the regulation of transcription and DNA damage repair. Herein, we use a suite of protein chemistry methods to explore how H2B-Ub stimulates hDot1L-mediated methylation of histone H3 on lysine 79 (H3K79me). By using semisynthetic ‘designer' chromatin containing H2B-Ub bearing a site-specifically installed photocrosslinker, here we report an interaction between a functional hotspot on ubiquitin and the N-terminus of histone H2A. Our biochemical studies indicate that this interaction is required for stimulation of hDot1L activity and leads to a repositioning of hDot1L on the nucleosomal surface, which likely places the active site of the enzyme proximal to H3K79. Collectively, our data converge on a possible mechanism for hDot1L stimulation in which H2B-Ub physically ‘corrals' the enzyme into a productive binding orientation.

## Abstract (web summary)

The ubiquitylation of histone H2B on lysine 120 is an important modification with roles in a diverse range of nuclear processes. Here, the authors use 'designer' chromatin to show that H2B-ub orients hDot1L into the correct position for activation.

## Graphical abstract

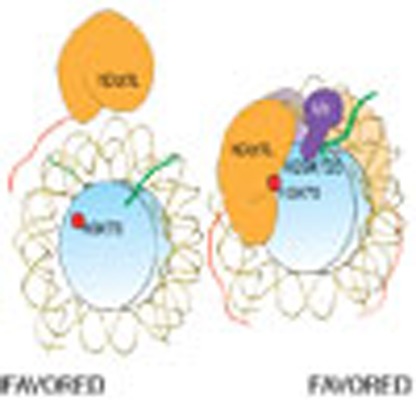

An emerging paradigm in epigenetic regulation is the functional crosstalk between histone post-translational modifications (PTMs), that is, where the presence of a pre-existing PTM on the chromatin template can positively or negatively regulate the local installation or removal of another PTM^1^. This interplay can operate in *cis*, where the crosstalk pair is located on the same histone protein, or in *trans* where the pair is located on different histones either within the same nucleosome or, in principle, spatially adjacent nucleosomes^1^. The trans-crosstalk phenomenon is exemplified by the interplay between H2B-Ub and methylation of histone H3 on lysine 79 (H3K79me)^2^,^3^,^4^, a PTM linked to a diverse set of nuclear processes including telomeric silencing^5^,^6^,^7^, transcriptional elongation^8^,^9^ and the DNA damage response^10^,^11^,^12^,^13^. Biochemical studies using reconstituted chromatin templates have shown that the methyltransferase responsible for installing H3K79me, Dot1 (termed hDot1L in humans), is directly stimulated by the presence of H2B-Ub^14^,^15^,^16^,^17^. The H3K79me/H2B-Ub crosstalk operates in an intranucleosomal fashion and requires a specific surface epitope on ubiquitin since other ubiquitin-like proteins with a similar size and structure to ubiquitin do not stimulate hDot1L activity when conjugated to H2B at the same site, that is, lysine 120 (refs 14, 15, 16). Structure-activity studies involving systematic alanine mutation of the ubiquitin surface have identified a specific surface epitope centred on Leu71/Leu73 required for hDot1L activation^18^. However, it remains unclear whether this ubiquitin ‘hotspot' engages the enzyme or the nucleosomal surface. Thus, several mechanisms remain possible for the activation of hDot1L by H2B-Ub, including recruitment of enzymatic function and any one of several allosteric activation processes^3^,^19^. Understanding the details of this trans-crosstalk mechanism is important since deregulated hDot1L activity, leading to hypermethylation of H3K79, is associated with certain hematopoietic malignancies^20^,^21^.

In this study, we used chemically defined ‘designer' chromatin^22^,^23^ to shed light on the hDot1L activation mechanism. We developed a synthetic route to H2B-Ub bearing a diazirine photocrosslinker within the aforementioned functional hotspot. This protein was incorporated into nucleosomes and crosslinking performed in the presence of hDot1L. Surprisingly, we find no evidence of an interaction between this critical surface epitope in ubiquitin and the methyltransferase. Rather, a strong crosslink is observed to histone H2A, which is localized to the N-terminal tail by mass spectrometry. Importantly, nucleosomes containing H2B-Ub but lacking the H2A tail show a reduced ability to activate hDot1L. Subsequent biochemical studies revealed that the presence of H2B-Ub changes both the number and binding orientation of hDot1L on nucleosomal substrates, with the N-terminus of H2A being required for this repositioning. Overall, our *in vitro* studies are consistent with a ‘corralling' type mechanism in which ubiquitin is positioned on the nucleosomal surface so as to occlude non-productive hDot1L binding orientations.

## Results

### Semi-synthesis of photocrosslinking conjugate 1 (H2BssUb*)

**Figure 1:**
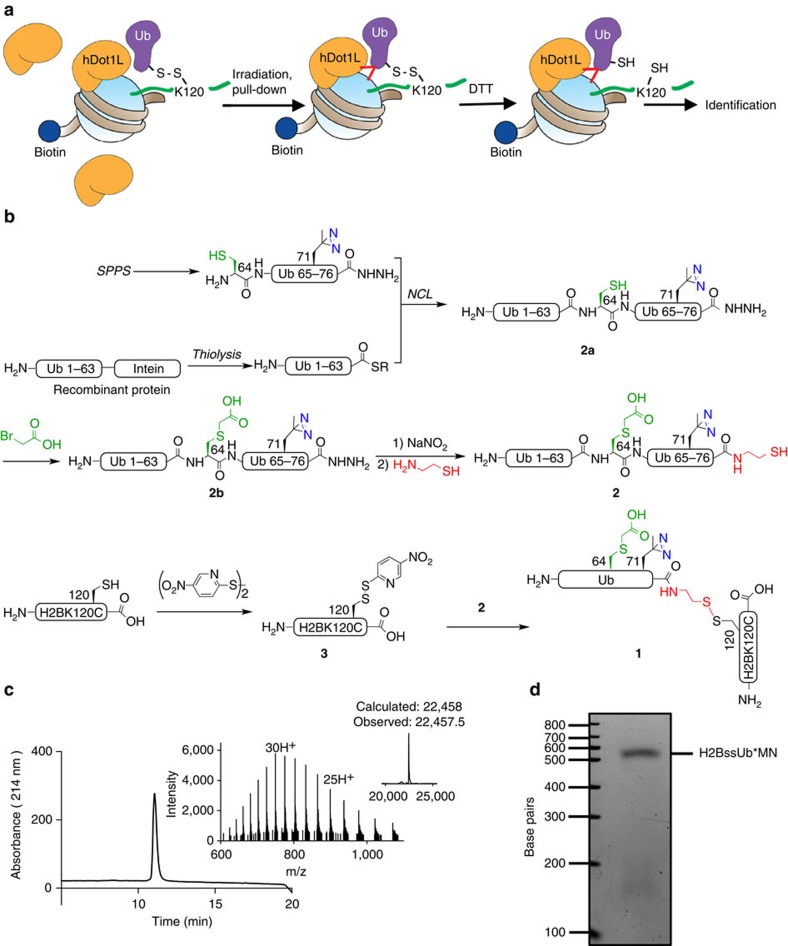
A targeted photocrosslinking strategy to identify the binding partner for the Leu71/Leu73 epitope on ubiquitin. (**a**) Schematic of crosslinking strategy using mononucleosomes containing H2BssUb* and the catalytic domain of hDot1L. Biotinylated mononucleosomes are incubated with the catalytic domain of hDot1L, ultraviolet irradiated and purified by streptavidin pull-down. Addition of dithiothreitol to the crosslinked mixture severs the ubiquitin-H2B disulfide bond. Ubiquitin interaction partners (hypothetical crosslinks are indicated by red lines) are subsequently identified by analysis of crosslinked species by immunoblotting or MS. (**b**) Semi-synthesis of H2BssUb* (**1**). A synthetic peptide corresponding to residues 64–76 of ubiquitin bearing L-photo-Leu at position 71, an E64C mutation and a C-terminal hydrazide moiety, was assembled using Fmoc-SPPS. The ubiquitin fragment Ub(1–63) was heterologously expressed in *E. coli* as an intein fusion and converted to an α-thioester by thiolysis *in vitro*. The two ubiquitin fragments were joined by NCL, forming full-length ubiquitin **2a**, which was reacted with bromoacetic acid to afford protein **2b**. Subsequent oxidation and ligation to cysteamine yielded ubiquitin analogue, **2**. Histone H2B bearing a K120C mutation was heterologously expressed in *E. coli*, and reacted with DTNP, generating the activated asymmetric disulfide species, **3**. In the final step, **2** and **3** were allowed to react at pH=6.9 to form the disulfide–linked H2BssUb*, protein **1**. (**c**) RP-HPLC and ESI-MS analysis of the final protein **1** (MW: 22,458 Da calculated, 22,457.5 Da observed). (**d**) Ethidium-bromide-stained native gel of reconstituted mononucleosomes containing H2BssUb*.

As a starting point for our studies, we focused on the recent identification of a discrete surface epitope on ubiquitin, centred on Leu71/Leu73, required for hDot1L activation^18^. Since it is unclear what this epitope engages to induce H3K79 methylation, we adopted a targeted photocrosslinking strategy in which Leu71 on ubiquitin was replaced by the isosteric, diazirine-containing amino acid, L-photo-leucine (Fig. 1a). A key feature of our strategy was the incorporation of an ubiquitin transfer step, which was expected to ease with the identification of the binding partner for the epitope (Fig. 1a). This led to the design of ubiquitylated construct **1** (also referred to as H2BssUb*) in which the photo-Leu-containing ubiquitin is linked to histone H2B via a cleavable disulfide linkage. Previous studies have shown the attachment of ubiquitin to H2B via this linkage does not affect hDot1L activation^16^. Thus, we imagined that use of a simple reduction step would reveal the ubiquitin binding partner through label transfer. Construct **1** was assembled in a convergent fashion using semisynthetic ubiquitin analogue **2** and activated H2B cysteine mutant **3** (Fig. 1b). Protein **2** was generated by expressed protein ligation using a recombinant, intein-derived α-thioester fragment corresponding to residues 1–63 of ubiquitin, and a synthetic peptide comprising the remainder of the ubiquitin sequence and containing both photo-Leu at position 71 and a Glu-to-Cys mutation at position 64, the latter to facilitate native chemical ligation. Importantly, the synthetic fragment contained an α-hydrazide moiety which enables the attachment of a thiol group at a later stage of the synthesis. Following the first ligation step, Cys64 was alkylated with bromoacetic acid to give an analogue of the native glutamate residue. Subsequently, the hydrazide-group was subjected to a one-pot oxidation, cysteamine ligation process to afford the desired C-terminal thiol-containing protein **2** (see Supplementary Fig. 1 for characterization of building blocks). Histone H2B in which Lys120 was mutated to cysteine was isolated from an *E. coli* overexpression system and then treated with 2,2'-dithiobis-(5-nitropyridine) to give the activated asymmetric disulfide protein **3**. Note, the cysteine at position 120 of H2B is the only cysteine within any of the four histones used in this study.

The necessary building blocks in hand, the desired semisynthetic construct, H2BssUb*, was generated using an established asymmetric disulfide exchange process^16^ and, following purification (Fig. 1c), incorporated into mononucleosomes and nucleosome arrays using standard procedures^17^,^24^ (Fig. 1d and Supplementary Fig. 2). As expected, H2BssUb* stimulated H3K79 methylation by the catalytic domain of hDot1L compared with non-ubiquitylated mononucleosomes (Supplementary Fig. 3). Moreover, activation of the methyltransferase was lost when H2BssUb* was pretreated with the reducing agent dithiothreitol, indicated the ubiquitin release mechanism needed for the crosslinking strategy was operational (Supplementary Fig. 3).

### Ubiquitylated H2B interacts with the N-terminal tail of H2A

**Figure 2:**
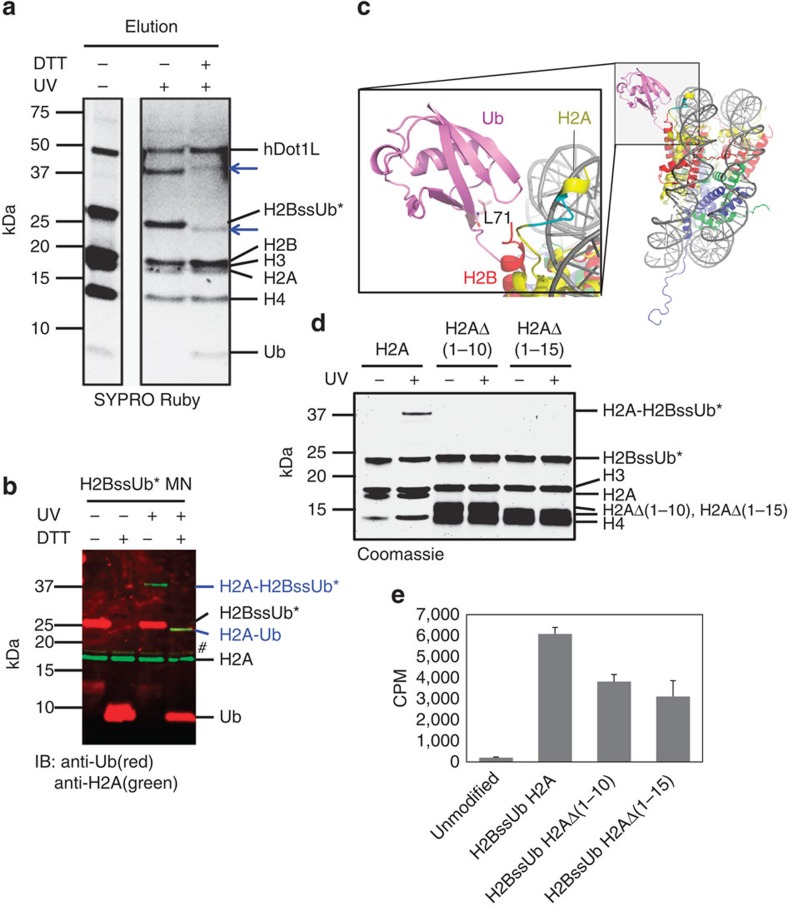
Ubiquitylated H2B interacts with the N-terminal tail of H2A. (**a**) SDS-PAGE analysis of the crosslinking reaction between biotinylated mononucleosomes containing H2BssUb* and a 40-fold molar excess of the catalytic domain of hDot1L. Reaction mixtures were purified by streptavidin pull-down before analysis—note, residual hDot1L remains non-covalently bound to mononucleosomes even after extensive washing. Addition of dithiothreitol, which cleaves the disulfide bond connecting Ub to H2B, led to the generation of a new species of lower molecular weight. Crosslinked species are indicated with blue arrows. (**b**) Western blot analysis of crosslinked species, with or without dithiothreitol treatment to complete the ubiquitin transfer step. Mononucleosomes containing H2BssUb* were UV irradiated and separated on a denaturing gel, followed by immunoblotting against ubiquitin (red) and H2A (green). The impurity (#) recognized by H2A antibody is H3 due to cross-reactivity of the antibody. Note, H2A-H2BssUb is recognized by anti-H2A antibody but not the anti-Ub antibody, which is likely due to epitope occlusion resulting from ubiquitin being sandwiched between H2A and H2B. (**c**) Composite structural model of a ubiquitylated mononucleosome (mononucleosome pdb code=1AOI, Ub pdb code=1UBQ). Ubiquitin, H2B and H2A are coloured in magenta, red and yellow, respectively. The N-terminal region of H2A, amino acids 7–10, is highlighted in cyan. Inset, close-up of the ubiquitin attachment site on H2B. (**d**) SDS-PAGE analysis of crosslinked reaction mixtures of mononucleosomes containing H2BssUb* and either full-length H2A, H2AΔ(1-10) or H2AΔ(1-15). (**e**) hDot1L methyltransferase assay on chemically defined mononucleosomes. Assays were performed on mononucleosomes with ^3^H-SAM and the catalytic domain of hDot1L. Quantification of methylation was performed by filter binding followed by liquid scintillation counting. Error bars indicate s.e.m. (*n*=3–4).

Next, we moved to the photocrosslinking experiments. Mononucleosomes containing H2BssUb* were incubated with the catalytic domain of hDot1L, ultraviolet irradiated and then resolved by non-reducing SDS-PAGE (Fig. 2a). This led to the appearance of a single new band at ∼40 kDa compared with the non-irradiated control. Note, we did not observe any crosslinking between H2BssUb* and nucleosomal DNA (Supplementary Fig. 4). The size of this new species was inconsistent with a crosslink between H2BssUb* and hDot1L (expected size 70 kDa), thus suggesting a crosslink between H2BssUb* and another core histone. Indeed, a repeat of the crosslinking experiment in the absence of the enzyme led to the generation of the same species and, furthermore, the same result was observed when H2BssUb* was incorporated into a 4-mer nucleosome array (Supplementary Fig. 5a). Note, the same crosslink species was observed using ‘designer' mononucleosomes in which the photo-Leu residue was incorporated at position 73 of ubiquitin, albeit with slightly lower efficiency (Supplementary Fig. 5b and Supplementary Methods). Immunoblotting of the gel indicated that the crosslinked species contained H2BssUb* and histone H2A (Fig. 2b, expected size 37 kDa). Addition of dithiothreitol to the crosslinked mixture, inducing the ubiquitin transfer step from H2B to the binding partner, led to the disappearance of the ∼40 kDa band and the appearance of a new species at ∼23 kDa that was confirmed to be H2A-Ub by immunoblotting and mass spectrometry (Fig. 2b and Supplementary Fig. 6, expected size 22 kDa).

Inspection of the mononucleosome crystal structure reveals that the ubiquitin attachment site on H2B (Lys120) is proximal to the H2A N-terminus^25^, making this region a prime candidate for the crosslink site (Fig. 2c). This idea was supported by tryptic digestion of the crosslinked species followed by liquid chromatography-tandem MS (LC-MS/MS) (Supplementary Fig. 6). To confirm this assignment, we repeated the crosslinking experiment with mononucleosomes containing truncated versions of H2A lacking either the first 10 (H2AΔ1-10) or 15 (H2AΔ1-15) amino acids (Supplementary Fig. 2). No crosslinked species was generated in either case (Fig. 2d), consistent with the idea that H2B-Ub interacts with the N-terminus of H2A. We also performed a hDot1L methyltransferase assay with ubiquitylated mononucleosomes containing truncated H2A and observed a ∼50% reduction in H3K79 methylation compared with control mononucleosomes containing full-length H2A (Fig. 2e). Thus, the interaction between H2B-Ub and H2A seems to be functionally important for hDot1L stimulation.

### H2B-Ub alters binding of hDot1L to mononucleosomes

**Figure 3:**
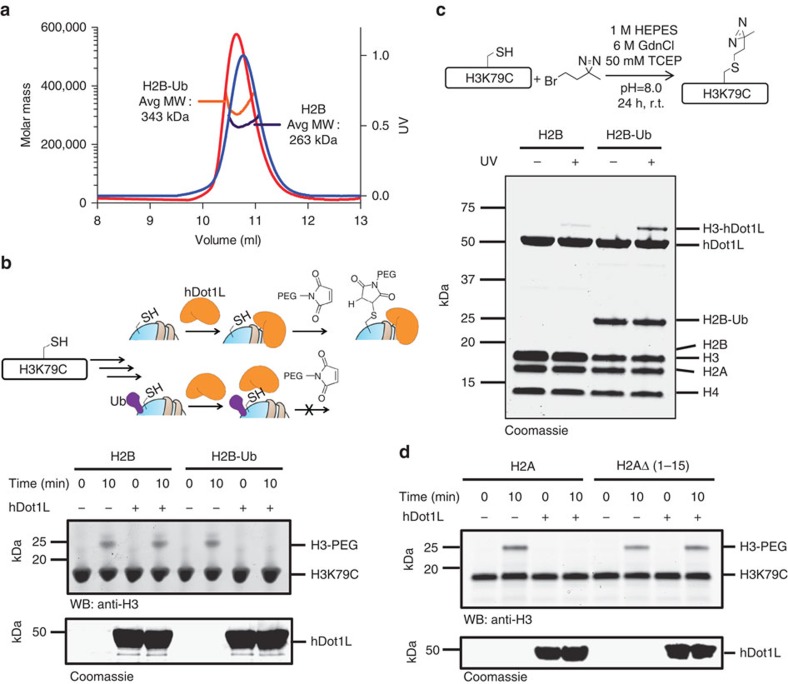
The presence of H2B-Ub alters the binding stoichiometry and the binding position of hDot1L and mononucleosomes. (**a**) Analysis of mononucleosome-hDot1L complexes by SEC-MALS. The UV traces of the ubiquitylated and non-ubiquitylated mononucleosome-hDot1L complexes are shown in red and blue, respectively. The orange and purple lines represent the corresponding measured molecular weights (MW) by MALS. Mw/Mn values of ubiquitylated and non-ubiquitylated mononucleosome-hDot1L complexes are 1.003, 1.001, respectively. Expected MWs (calculated based on SEC-MALS analysis of individual components) are as follows: for unmodified mononucleosome:hDot1L complex with 1:1 stoichiometry=267 kDa, 1:2=313 kDa; for ubiquitylated mononucleosome:hDot1L complex with 1:1 stoichiometry=304 kDa, 1:2=350 kDa. (**b**) H3K79C accessibility in the presence or absence of hDot1L. Top; schematic of the foot-printing assay used to assess the ability of hDot1L to protect the *S*-alkylation of H3K79C by PEG-maleimide as a function of H2B ubiquitylation. Bottom; western blot analysis of PEGylation assay. (**c**) H2B ubiquitylation stimulates Dot1L binding to H3K79. Top; schematic of the photocrosslinking strategy used. H3K79C is alkylated to yield a diazirine-containing side chain at K79. Biotinylated mononucleosomes containing H3K79C[diazirine] are then used in photocrosslinking studies with hDot1L. Bottom; SDS-PAGE analysis of indicated reaction mixtures following streptavidin pull-down. (**d**) hDot1L binding to ubiquiylated mononucleosomes as a function of H2A N-terminal tail. Indicated ubiquitylated mononucleosomes harbouring the H3K79C were incubated with hDot1L and then treated with PEG-maleimide followed by analysis by western blotting.

We next turned to the question of how the interaction between H2B-Ub and H2A might contribute to the regulation of hDot1L activity. It has been shown previously that hDot1L binds chromatin regardless of the presence or absence of H2B-Ub^14^,^26^,^27^. We confirmed this observation using pull-down experiments using biotinylated mononucleosomes (Supplementary Fig. 7). Analysis of the resulting complexes by size-exclusion chromatography coupled to multi-angle light scattering (SEC-MALS) yielded a more surprising result, namely that unmodified mononucleosomes bind a single copy of hDot1L, whereas mononucleosomes containing H2B-Ub bind two copies of the enzyme (Fig. 3a). These binding stoichiometries were confirmed by SDS-PAGE analysis of the SEC fractions using ratiometric standards (Supplementary Fig. 8). Note, the H2B-Ub used in these and, indeed, all subsequent studies contained a stable isopeptide linkage^15^ rather than the cleavable disulfide linkage used in the initial crosslinking studies.

### H2B-Ub is required for productive positioning of hDot1L

The SEC-MALS data imply that the presence of H2B-Ub markedly changes the nature of the binding interaction between hDot1L and the nucleosome, presumably leading to a repositioning of the enzyme active site so as to promote H3K79 methylation. To explore this idea, we developed a foot-printing assay based on the ability to *S*-alkylate mononucleosomes bearing a unique cysteine residue at position 79 of H3 (Fig. 3b). Addition of a polyethylene glycol (PEG)-maleimide reagent to mononucleosomes containing the H3K79C mutant led to the generation of the expected PEGylated H3 adduct (Fig. 3b, lanes 1 and 2). This adduct was also generated when using mononucleosomes containing H2B-Ub, indicating that ubiquitin itself does not impede the reaction. By contrast, we observed a remarkable difference in reactivity in the presence of hDot1L; non-ubiquitylated mononucleosomes were alkylated to a similar extent as the enzyme-free control, whereas H2B-Ub containing mononucleosomes were completely protected from PEGylation (Fig. 3b, compare lanes 3 and 4 with lanes 7 and 8). The altered binding orientation of hDot1L on the mononucleosome surface, as a function of ubiquitylation, was further examined through photocrosslinking. In this case, we attached a diazirine moiety to the sulfhydryl group of the H3K79C mutant using 3-(2-bromoethyl)-3-methyl-3H-diazirine (Fig. 3c). Mononucleosomes containing this alkylated histone H3 analogue were incubated with hDot1L, irradiated and analysed by SDS-PAGE. This revealed the generation of a new species whose migration was consistent with an H3-hDot1L crosslink. This assignment was confirmed by MS analysis (Supplementary Fig. 9). Importantly, the presence of H2B-Ub in the mononucleosomes led to a substantial increase in the amount of this H3-hDot1L crosslink, which provides further evidence for a repositioning of the enzyme on the mononucleosome surface upon ubiquitylation of H2B. Finally, to probe the role of the H2A N-terminus in the regulation of hDot1L binding, we repeated the PEGylation assay using ubiquitylated mononucleosomes containing truncated H2A (Fig. 3d). In stark contrast to the case with full-length H2A where no PEGylation was found in the presence of hDot1L, we now observed the generation of the H3-PEG adduct. Thus, the interaction between H2B-Ub and H2A is required for hDot1L to assume a productive binding orientation on the mononucleosome.

## Discussion

The crosstalk between H3K79me and H2B-Ub was first appreciated in genetic studies of yeast^2^,^3^,^4^ and later shown to operate via direct stimulation of Dot1 in biochemical experiments that used ‘designer' chromatin^14^,^15^,^16^. The molecular mechanism of this stimulation has, however, remained enigmatic. The data presented herein shed considerable light on this issue and converge on a mechanism in which the presence of H2B-Ub directly alters the binding of hDot1L on the nucleosomal surface. In the course of this investigation we identified, and confirmed the functional importance of, an interaction between a previously identified hotspot on the ubiquitin surface and the N-terminal tail of H2A. In geometric terms, this interaction is entirely reasonable given the juxtaposition of the H2B C-terminus and H2A N-terminus in the nucleosome structure, especially since the epitope on ubiquitin is close to the Lys120 attachment site (Fig. 2c). Our studies implicate the first ten residues of H2A tail in the ubiquitin interaction. It is worth noting that the sequence of this region of H2A differs from the analogous region in histone H4 by only a single residue, the insertion of a glutamine residue in the former (Supplementary Fig. 10). While we saw no evidence for an interaction between H2B-Ub and H4 in the present studies (even in nucleosome arrays) it is nonetheless conceivable that such a binding partner switch might occur under some situations perhaps driven by differential modification of the H2A/H4 tails or the presence of other binding partners.

**Figure 4:**
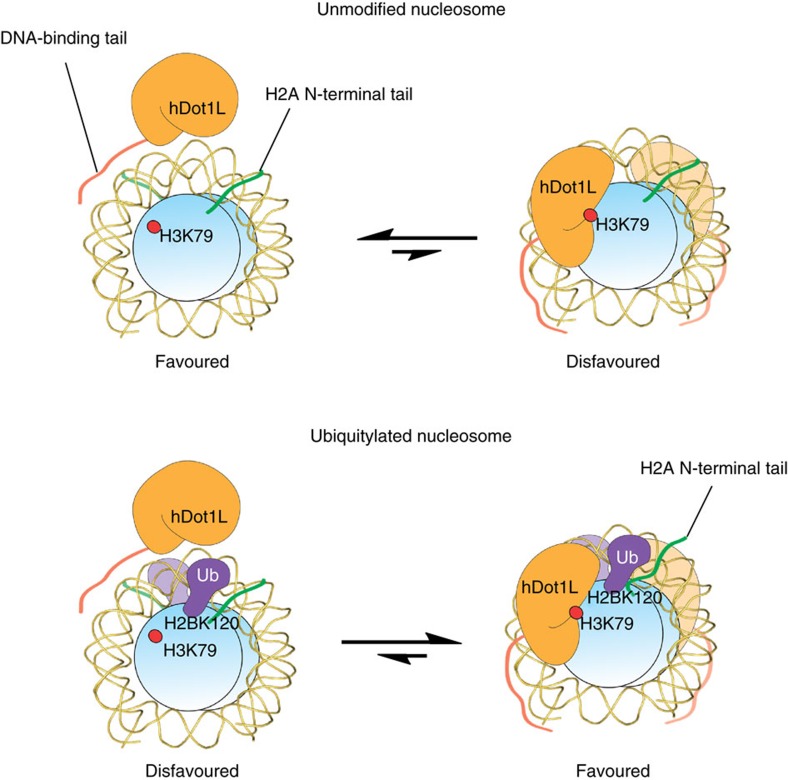
Proposed ‘corralling' mechanism of hDot1L activation by H2B-Ub. Core histones, hDot1L and ubiquitin are coloured in blue, yellow and purple, respectively. The DNA-binding region of hDot1L is illustrated in orange, and the N-terminal tail of H2A is shown in green. Top, binding model of hDot1L and an unmodified nucleosome. In this case, a non-productive 1:1 enzyme-substrate complex is favored. Bottom, binding model of hDot1L with a ubiquitylated nucleosome. The presence of H2B-Ub in the nucleosome physically obstructs the formation of non-productive enzyme-substrate complexes and corrals hDot1L on the nucleosomal surface to its catalytically productive position.

We discovered that the presence of H2B-Ub alters the binding stoichiometry between hDot1L and mononucleosomes (Fig. 3a). That two copies of the enzyme would engage ubiquitylated mononucleosomes is not surprising given that the core particle contains two copies of each histone. This finding is also consistent with previous enzymatic data that showed a 1:1 stimulation of H3K79me by H2B-Ub^15^. Thus, we imagine that each face of the ubiquitylated mononucleosome engages a copy of hDot1L. More puzzling is our observation that unmodified mononucleosomes bind to only a single copy of hDot1L. The enzyme is known to bind chromatin via a basic stretch of amino acids adjacent to the catalytic domain^26^ (Fig. 4). Presumably, this region engages DNA in a rather non-specific manner and so it is unclear why multiple copies of the enzyme do not bind to unmodified mononucleosomes, particularly since we know this is possible in the context of ubiquitylated mononucleosomes. Conceivably, hDot1L might preferentially bind to unmodified mononucleosomes in a manner that is unproductive catalytically, for example, at or near the diad axis, which also sterically prevents higher binding stoichiometry given the large size of hDot1L. Presumably, the basal level of hDot1L activity observed on unmodified chromatin reflects infrequent excursions to a productive binding orientation, perhaps promoted by the previously postulated interaction between the enzyme and a basic patch on the H4 tail^28^,^29^.

Integrating the available biochemical data on the system^14^,^15^,^16^,^18^, including the studies described herein, we favour a hDot1L activation mechanism in which the presence of H2B-Ub in the nucleosome physically obstructs the formation of non-productive enzyme-substrate complexes that dominate in the absence of the PTM (Fig. 4)^18^,^30^. The interaction between ubiquitin and the H2A tail likely helps constrain the possible orientations of the PTM on the mononucleosome surface, which in turn restricts the binding modes possible for the enzyme, increasing the chance of a productive complex. It is worth noting that H2B-Ub still stimulates hDot1L activity in the absence of the H2A tail, albeit to a significantly reduced degree compared with when it is present. Conceivably, this might reflect other interactions between ubiquitin and the nucleosomal surface not captured in our crosslinking experiments. Regardless, the proposed ‘corralling' model explains why mutation of the ubiquitin hotspot^18^, or use of other ubiquitin-like proteins^15^,^16^, does not activate hDot1L; in these cases, the interaction between Ub and the nucleosomal surface is lost and so the now less constrained PTM does not provide as effective a steric barrier to guide productive enzyme binding. We note that this activation model does not include a specific interaction between ubiquitin and hDot1L. This is based on our previous mutational data, which did not identify any essential surface of ubiquitin beyond the Leu71/Leu73 hotspot that we now believe engages the nucleosome^18^. Thus, we imagine the remainder of ubiquitin acts as steric bulk. We note that Leu71/Leu73 hotspot on ubiquitin is also required for the stimulation of the H3K4 methyltransferase, Set1, by H2B-Ub^18^. Thus, an analogous steric mechanism may also contribute to this activation process. Future studies will address this intriguing possibility.

In conclusion, we have deployed a suite of protein chemistry methods to dissect the mechanism of hDot1L activation by H2B-Ub, ultimately arriving at a model based on alternate enzyme binding modes as a function of the PTM. Beyond the current insights, we imagine that several of the methods introduced herein, which exploit the ability to site-specifically introduce crosslinkers into PTMs and probe cysteine accessibility, will have utility for studying the engagement of other chromatin modifiers with their substrates.

## Methods

### EPL to generate ubiquitin derivative 2

Ubiquitin containing a photocrosslinker at position 71 was prepared by protein semi-synthesis. N-α-Fmoc-photoleucine was synthesized from L-Photo-Leucine (L*) as previously described^31^. The C-terminal ubiquitin fragment Ub(64-76)E64C/L71L* was synthesized on solid phase as a peptide hydrazide^32^ using a standard fluorenylmethoxycarbonyl Nα protection strategy on an automated peptide synthesizer. Recombinant Ub(1–63) was produced in *E. coli* as a fusion to the NPU intein^18^ and converted into a reactive α-thioester by thiolysis with sodium 2-mercaptoethanesulfonate. To join the two fragments, 2.0 μmol of Ub(64–76)E64C/L71L* peptide hydrazide (2.9 mg) was dissolved in TCEP ligation buffer (6 M guanidinium chloride, 100 mM TCEP, 300 mM phosphate, pH 7.5 at room temperature) to a final concentration of 2 mM and the pH readjusted to 7.5. In parallel, a total of 1.0 μmol of the Ub(1–63) α-thioester was dissolved in mercaptophenylacetic acid ligation buffer (6 M guanidinium chloride, 300 mM phosphate, 100 mM mercaptophenylacetic acid, pH 7.5) to a final concentration of 1 mM. The two solutions were combined and ligation allowed to proceed overnight after which the product was purified by semipreparative RP-HPLC using a gradient of 25–55% B in H_2_O containing 0.1% TFA over 45 min (solvent B=90% CH_3_CN, 10% H_2_O, 0.1% TFA). The identity of purified protein **2a** was verified by ESI-MS (calculated MW: 8,564 Da, observed MW: 8,564.5 Da; Supplementary Fig. 1). The ligation junction was then masked by conversion of Cys64 into a glutamate analogue. In all, 1.5 μmol of protein **2a** and 15 μmol of bromoacetic acid were dissolved in alkylation buffer (6 M guanidinium chloride, 300 mM phosphate, pH 8.0) and the alkylation reaction allowed to proceed overnight at which time it was deemed complete by RP-HPLC and ESI-MS (**2b**, calculated MW: 8,622 Da, observed MW: 8,621.6 Da). This product was directly converted into thiol-containing derivative **2** upon oxidation of the acyl hydrazide moiety. The reaction mixture was cooled to −10 °C in an ice/NaCl bath and the pH adjusted to 3.0. NaNO_2_ was added dropwise with mixing from a 200 mM stock solution to a final concentration of 20 mM. The reaction was allowed to proceed at −10 °C for 45 min. The solution was subsequently brought to room temperature, the pH adjusted to 7.0 and cysteamine (10 mg, 90 μmol) added. The ligation reaction was monitored by RP-HPLC and ESI-MS and went to completion within an hour. Fresh TCEP was added before purification by semipreparative RP-HPLC using a gradient of 25–55% B over 45 min. The final product protein **2** was analysed by RP-HPLC and ESI-MS (calculated MW: 8,668 Da, observed MW: 8,667.6 Da; yield: 25%; Supplementary Fig. 1).

### Generation of histone H2B derivative 3

Recombinant H2B containing a Cys residue at position 120 was converted into an asymmetric disulfide as previously described with minor modifications^16^. DTNP (2.0 mg, 6.4 mmol), dissolved in 500 μl of a 3:1 (v/v) acetic acid:water mixture, was added to H2BK120C (3.0 mg, 0.2 μmol). The reaction was allowed to proceed for 12 h at 25 °C, before purification by semipreparative RP-HPLC using a 42-48% B gradient at 40 °C over 45 min, yielding pure H2BK120C[5-nitro-2-pyridinesulfenyl] (pNpys) disulfide product (calculated MW: 13,946 Da, observed MW: 13,946.0 Da; Supplementary Fig. 1).

### Generation of photocrosslinking conjugate 1 (H2BssUb*)

H2B-ubiquitin conjugate **1** was synthesized from **2** and **3** by disulfide exchange using an established protocol^16^. In a typical reaction, 1 equivalent of protein **3** (1.5 mg, 0.1 μmol) and 2.1 equivalents of protein **2** (2.0 mg, 0.2 μmol) were dissolved in 625 μl of reaction buffer consisting of 1 M HEPES, 6 M guanidinium chloride, pH 6.9. The reaction was incubated for 1 h at 25 °C with continuous shaking, followed by purification of the product by semipreparative RP-HPLC with a 45–60% B gradient over 45 min. The identity of ligation product **1** was verified by ESI-MS (calculated MW: 22,458 Da, observed MW: 22,457.5 Da; Fig. 1c).

### Synthesis of H3K79C[diazirine]

A photocrosslinking derivative of histone H3 was prepared by cysteine alkylation. Recombinant histone mutant H3K79C (1.0 μmol, 15 mg) was dissolved in ligation buffer (1 M HEPES, 6 M guanidinium chloride, 50 mM TCEP, pH 8.0) to a final concentration of 10 mg ml^−1^. A total of 150 equivalents of 3-(2-bromoethyl)-3-methyl-3H-diazirine (150 μmol, Enamine Chemicals) were added to the protein solution and the pH was readjusted to 8.0. The ligation reaction was incubated overnight at room temperature and the product purified by semipreparative RP-HPLC using a gradient of 40–60% B over 45 min. The identity of the ligation product was verified by ESI-MS (calculated MW: 15,296 Da, observed MW: 15,295.4 Da; Supplementary Fig. 11).

### Histone octamer refolding

Histone octamers were formed as follows^17^. Briefly, individual histones (characterized by ESI-MS, Supplementary Fig. 13) were dissolved in unfolding buffer (6 M guanidinium chloride, 20 mM Tris-HCl, pH 7.5) at ∼4 mg ml^−1^. Equimolar amounts of H3 and H4 were mixed along with 1.05 equivalents of H2A and H2B, and the solution was diluted to ∼1 mg ml^−1^ with unfolding buffer. The resulting mixture was dialyzed into refolding buffer (2 M NaCl, 10 mM Tris-HCl, 1 mM EDTA, pH 7.5), and subsequently purified using a Superdex 200 10/300 size-exclusion column, eluted with refolding buffer. Octamer quality was analysed by SDS-PAGE. Octamer samples were stored at −20 °C in 50% glycerol.

### Nucleosome reconstitution

Mononucleosomes and nucleosomal arrays were prepared by a gradual dialysis of DNA and histone octamers (ratios were empirically optimized) into a low-salt buffer^17^. Purified octamers with DNA (unlabelled 147 bp dsDNA corresponding to the 601 targeting sequence, or biotinylated 147 bp dsDNA) were combining in high-salt buffer (20 mM Tris-HCl, 2 M NaCl, pH 7.5). The mixture was dialyzed (3,500-Da cutoff) against 200 ml nucleosome reconstitution buffer (10 mM Tris-HCl, 1.4 M KCl, 0.1 mM EDTA, pH 7.5) for 1 h at 4 °C. Subsequently, 330 ml of nucleosome reconstitution end buffer (10 mM Tris-HCl, 10 mM KCl, 0.1 mM EDTA, pH 7.5) was added to the solution at a rate of 1 ml min^−1^ using a peristaltic pump, followed by two final dialysis steps against nucleosome reconstitution end buffer (1 h and overnight). 4-mer nucleosome arrays were formed similarly by using DNA segments containing four repeats of the 601 nucleosome positioning sequence separated by 30-bp linkers. Nucleosome integrity was verified by separation on a Criterion 5% TBE gel run in 0.5 × TBE, followed by staining with ethidium bromide.

### Preparation of the catalytic domain of hDot1L

The cysteine-less catalytic domain of hDot1L, hDot1L(1–416)C44S/C74S/C178S (referred as hDot1L in the main text), was prepared as follows^18^. Briefly, *E. coli* BL21(DE3) cells, transformed with pET30a-His-Sumo-hDot1L(1–416), were grown in 6 l LB media at 37 °C until mid-log phase, and production of the hDot1L(1–416) His-Sumo fusion protein was induced by the addition of 0.5 mM IPTG at 18 °C for 18 h. After collecting the cells by centrifugation at 6,000*g* for 15 min, the cell pellet was resuspended in lysis buffer (25 mM Tris-HCl, 150 mM NaCl, 1 × protease inhibitor, pH 7.8) and lysed by passage through a French press. The insoluble material was removed by centrifugation at 26,000*g* for 30 min. The supernatant was filtered and incubated overnight at 4 °C with Ni-NTA beads (5 ml) pre-equilibrated with lysis buffer. The beads were washed with 50 ml of wash buffer (25 mM Tris-HCl, 150 mM NaCl, pH 7.8) containing increasing amounts of imidazole (25–100 mM), and eluted with 50 ml of the same buffer containing 250 mM imidazole. Pure fractions were combined and digested with Ulp1 at 4 °C for 30 min. Following digestion, the protein solution was loaded onto a HiTrap SP FF 5-ml column. After washing with 10 ml start buffer (20 mM Tris-HCl, 10 mM NaCl, pH 7.5), untagged catalytic domain of hDot1L was eluted with a 50 ml linear gradient from start buffer to end buffer (20 mM Tris-HCl, 1 M NaCl, pH 7.5). Fractions deemed pure by SDS-PAGE were pooled and further purified using a Superdex 200 10/300 column, eluted with elution buffer (25 mM Tris-HCl, 150 mM NaCl, pH 7.8; Supplementary Fig. 12). Pure fractions were concentrated and stored at −80 °C in 20% glycerol.

### Methyltransferase assays

Mononucleosomes (1.0 pmol) were combined with the catalytic domain of hDot1L (0.1 pmol) in 10 μl of assay buffer (25 mM Tris-HCl, 150 mM NaCl, pH 7.8). To initiate the reaction, ^3^H-S-adenosyl methionine (0.5 μCi, 66.7 pmol) was added and the reaction was allowed to proceed at 30 °C for 20 min. Aliquots (7 μl) of the reaction mixture were then spotted on Whatman p81 filter paper and the filters washed 3 times for 10 min with 0.2 M sodium carbonate, pH 9 and dried. Filter papers were added to 3.5 ml Ultimate Gold Liquid Scintillation Cocktail (Beckman Coulter). The samples were vortexed for 10 s and counted using a Microbeta2 Liquid scintillation counter (Perkin Elmer).

### Photocrosslinking assay with hDot1L

Biotinylated mononucleosomes (20 pmol) were combined with hDot1L (800 pmol) in 25 μl assay buffer (25 mM Tris-HCl, 150 mM NaCl, pH 7.8) containing 1 mM SAH, and incubated at 4 °C for 30 min. The mixtures were irradiated under a 120 watt ultraviolet lamp at 365 nm at room temperature for 25 min and subsequently bound to streptavidin magnetic beads. The beads were washed extensively with washing buffer (25 mM Tris-HCl, 150 mM NaCl, pH 7.8) and eluted after incubation with elution buffer (25 mM Tris-HCl, 2 M NaCl, pH 7.8). Note, in some cases dithiothreitol (100 mM) was included in the elution buffer to cleave the disulfide connecting ubiquitin to H2B. Eluted proteins were separated by non-reducing SDS-PAGE and visualized by SYPRO Ruby protein gel stain (Thermo Scientific) or GelCode Blue Safe Protein Stain (Thermo Scientific).

### Western blot and LC-MS/MS analysis of crosslinked bands

Mononucleosomes (20 pmol) in 20 μl of assay buffer (25 mM Tris-HCl, 150 mM NaCl, pH 7.8) were irradiated under a 120 watt ultraviolet lamp at 365 nm at room temperature for 25 min. The crosslinked mixtures were then separated by non-reducing SDS-PAGE and visualized by GelCode Blue Safe Protein Stain (Thermo Scientific). The crosslinked band was cut from the gel, digested with trypsin using an established protocol^33^ and subjected for LC-MS/MS analysis. LC-MS/MS analyses were performed on a reversed-phase nano-UPLC-MS platform, containing an Easy nLC Ultra 1000 nano-UPLC system that is coupled to an Orbi Elite mass spectrometer (Thermo Fisher Scientific) and equipped with a Flex Ion Source (Proxeon Biosystems, Odense, Denmark). Alternatively, western blotting was performed using antibodies against H2A (Abcam, ab58550, 1:200 dilution) and against ubiquitin (Abcam, ab52664, 1:500 dilution). Uncropped versions of blots appear in Supplementary Fig. 14.

### Size-exclusion chromatography coupled to multi-angle light scattering

Mononucleosomes (300 pmol) were combined with the catalytic domain of hDot1L (8.5 nmol) in 400 μl of MALS buffer (25 mM Tris-HCl, 150 mM NaCl, pH 7.8) and incubated at 4 °C for 30 min. The mixtures were purified using a Superdex 200 10/300 column, eluted with MALS buffer and analysed by on-line Wyatt MALS detectors. The average molecular weight of complexes was determined using the light scattering software Astra 6. Collected peak fractions were also analysed by SDS-PAGE alongside a series of H4 and hDot1L standards (concentration determined by integration of absorbance peak at 214 nm on RP-HPLC). Quantification of mononucleosomes and hDot1L in the complex was carried by densitometry using H4 or hDot1L calibration curves, respectively.

### Cysteine accessibility assays

Mononucleosomes (5 pmol) were combined with the catalytic domain of hDot1L (50 pmol) in 20 μl of PEGylation buffer (25 mM Tris-HCl, 150 mM NaCl, 4 mM EDTA, pH 7.8), and incubated at 30 °C for 10 min. To initiate the reaction, maleimide PEG5K (Nanocs) was added from a stock solution (1 mM maleimide PEG5K in PEGylation buffer) to a final concentration of 200 μM. The reaction mixtures were incubated at 30 °C for 15 min. and then quenched on ice through the addition of 1 μl dithiothreitol solution (100 mM dithiothreitol in PEGylation buffer). The samples were separated by SDS-PAGE, transferred to a PVDF membrane and visualized by blotting for H3 (Abcam, ab18521, 1:500 dilution).

## Additional information

**How to cite this article:** Zhou, L. *et al.* Evidence that ubiquitylated H2B corrals hDot1L on the nucleosomal surface to induce H3K79 methylation. *Nat. Commun.* 7:10589 doi: 10.1038/ncomms10589 (2016).

## Supplementary Material

Supplementary Information Supplementary Figures 1-14, Supplementary Methods and Supplementary References
