# Supplementary material for: Evidence that ubiquitylated H2B corrals hDot1L on the nucleosomal surface to induce H3K79 methylation
Source: Nat Commun. 2016 Feb 2;7:10589. doi: 10.1038/ncomms10589 (PMC4740876; doi:10.1038/ncomms10589)
Supplement: Supplementary Information — Supplementary Figures 1-14, Supplementary Methods and Supplementary References [file ncomms10589-s1.pdf]

Supplementary Figures

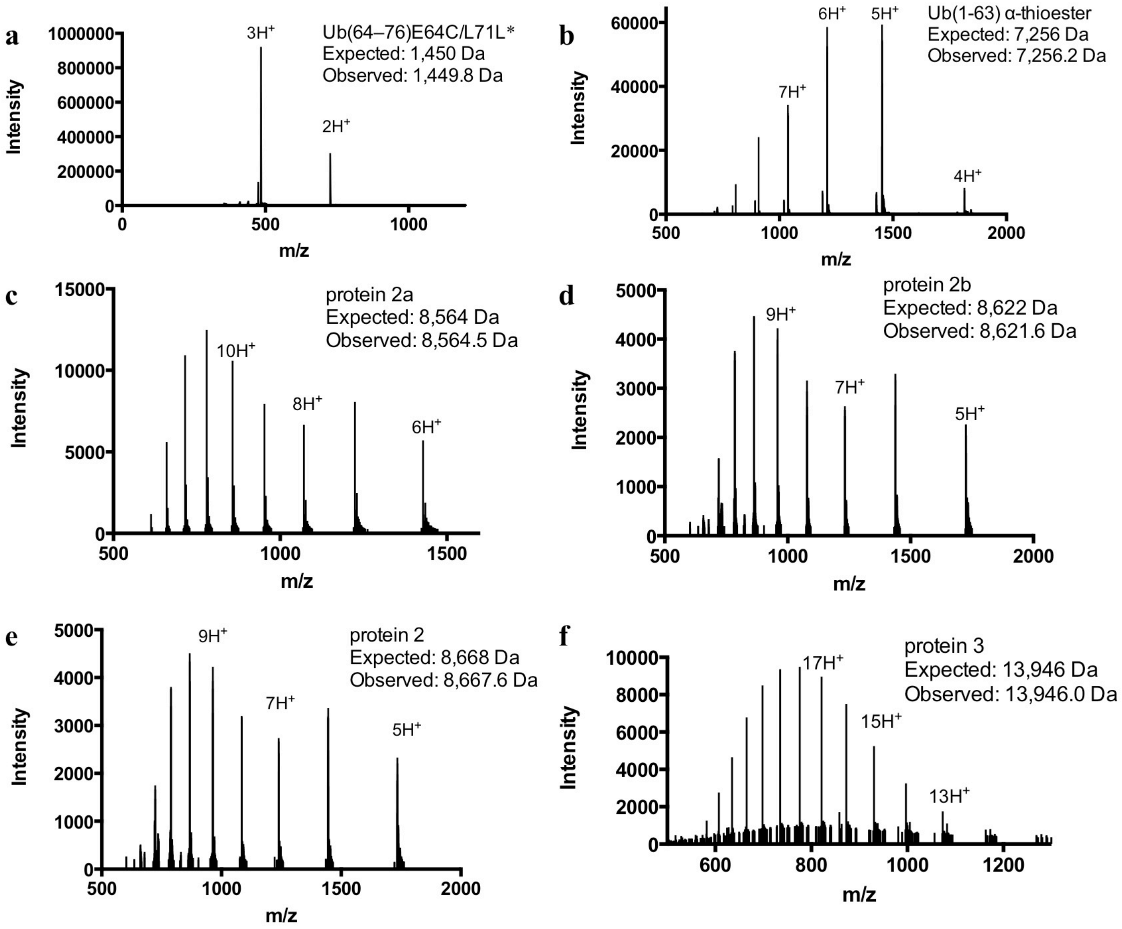

Supplementary Figure 1: Characterization of semisynthesis building blocks by ESI-MS. (**a**) ESI-MS characterization of Ub(64–76)E64C/L71L*. (**b**) ESI-MS characterization of Ub(1-63) α-thioester. (**c**) ESI-MS characterization of protein 2a. (**d**) ESI-MS characterization of protein 2b. (**e**) ESI-MS characterization of protein 2. (**f**) ESI-MS characterization of protein 3.

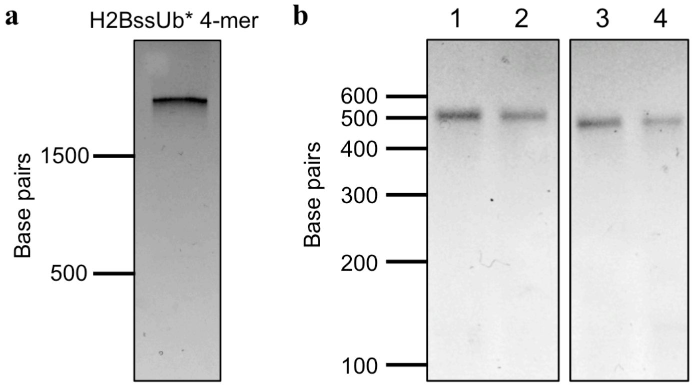

Supplementary Figure 2: Characterization of reconstituted 4-mer nucleosome arrays and MNs. (**a**) Ethidium-bromide-stained native gel of reconstituted 4-mer nucleosome arrays containing H2BssUb*. (**b**) Ethidium-bromide-stained native gel of reconstituted MNs containing both H2BssUb* and truncated H2A. Lanes correspond to MNs containing: H2AΔ(1-10) and H2BssUb* (lane 1), H2AΔ(1-10) and H2BssUb (lane 2), H2AΔ(1-15) and H2BssUb* (lane 3), H2ΔA(1-15) and H2BssUb (lane 4).

a

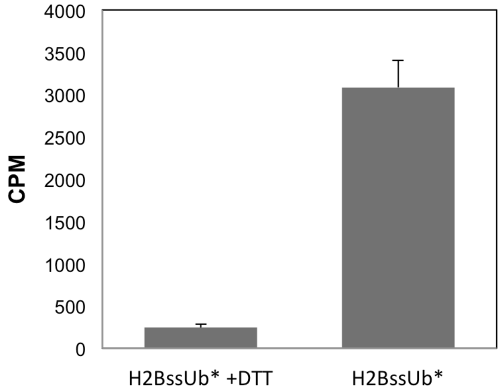

b

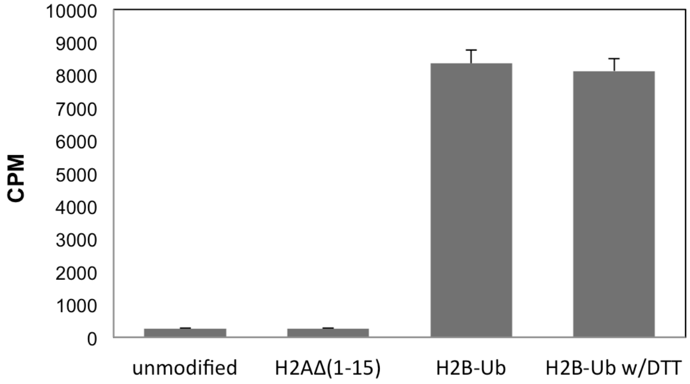

Supplementary Figure 3: hDot1L methyltransferase assay on reconstituted mutant MNs. Assays were performed on mutant MNs, some with DTT pre-treatment. Quantification of methylation was performed by filter binding followed by liquid scintillation counting. Error bars, s.e.m. (n=2-4). (**a**) Activation of hDot1L was lost when MNs containing H2BssUb* are pretreated with 100 mM DTT (with a final concentration of 50mM in methyltransferase assays). (**b**) Deletion of H2A N-terminus did not impact methylation of non-ubiquitylated nucleosomes (lanes 1 and 2) and addition of 50 mM DTT to ubiquitylated nucleosomes (containing a stable isopeptide linkage between Ub and H2B) had no impact in hDot1L activity.

a

b

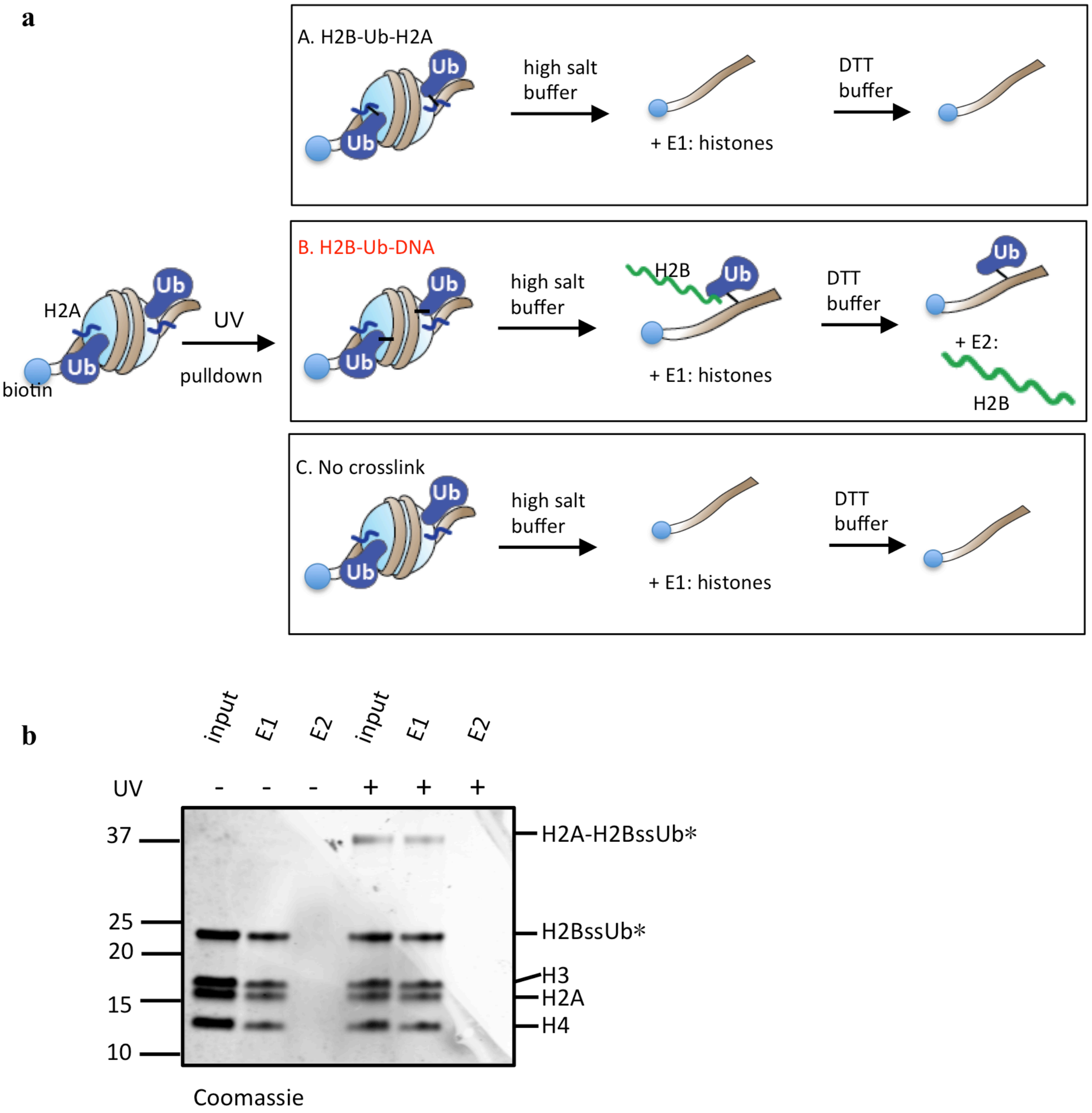

Supplementary Figure 4: No crosslinking between H2BssUb* and nucleosomal DNA was observed. (**a**) Schematic view of a crosslinking experiment to detect H2BssUb*-DNA crosslinking. Biotinylated MNs containing H2BssUb* were UV irradiated at room temperature for 25min and let bind to magnetic streptavidin beads. After washing the beads, histones were first eluted with high salt elution buffer (25mM Tris-HCl, 2M NaCl, pH 7.8), yielding elution 1 (E1). Beads were then eluted with DTT elution buffer (25mM Tris-HCl, 150mM NaCl, 100mM

DTT, pH 7.8), yielding elution 2 (E2). In the case that H2BssUb* crosslinks to nucleosomal DNA, H2BssUb* would remain bound on the beads after first elution and H2B would be cleaved off the beads during second elution. (**b**) Stained denaturing gel of crosslinking experiment to detect H2BssUb*-DNA crosslinking. No protein was observed in E2 with irradiation, indicating that there was no detectable crosslinking between H2BssUb* and nucleosomal DNA.

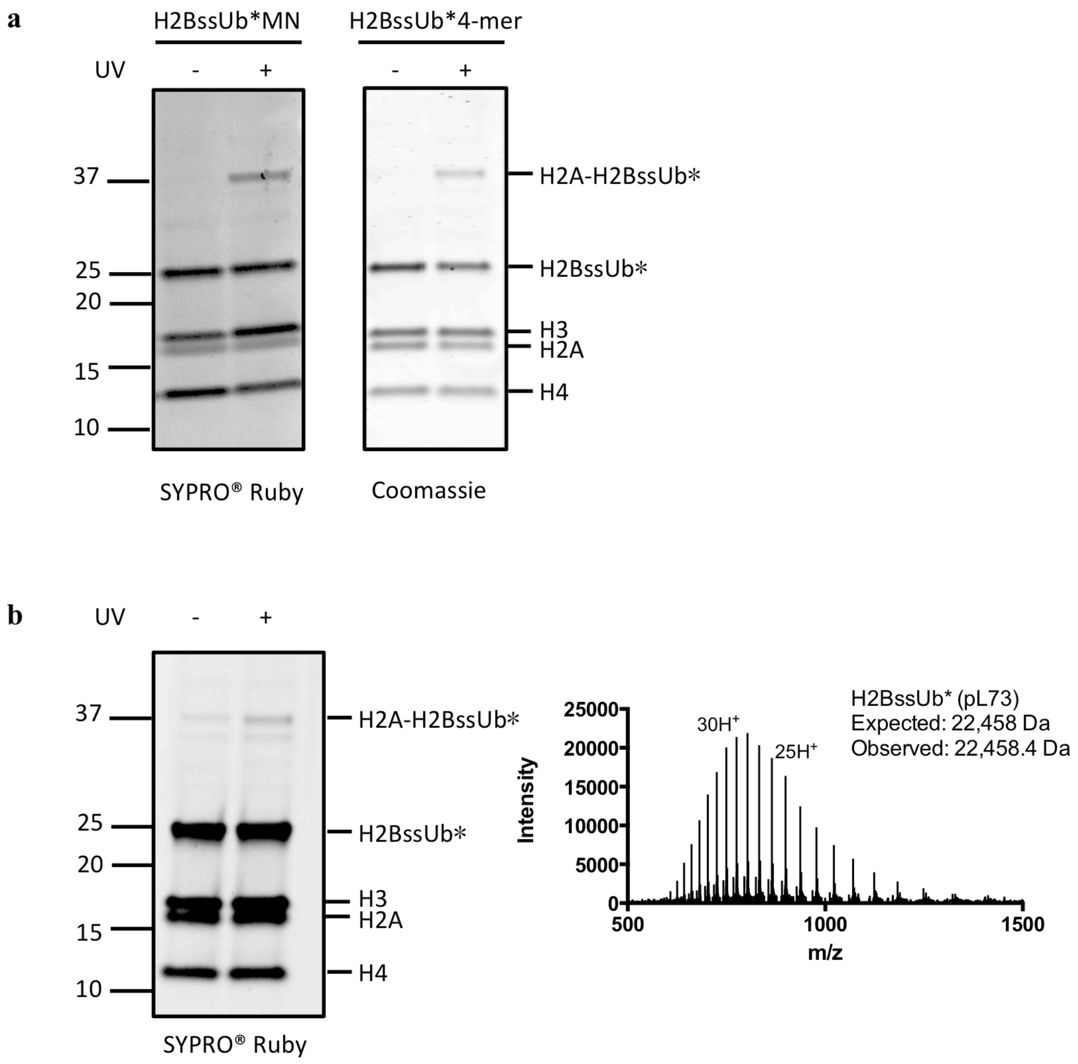

Supplementary Figure 5: Crosslinking experiment using H2BssUb* containing MNs or 4-mer arrays in the absence of hDot1L generates the same crosslinked species. (**a**) MNs or 4-mer arrays containing H2BssUb* (crosslinker incorporated at position 71 of ubiquitin) were UV irradiated at room temperature for 25min, separated on a denaturing gel and stained. In both cases, a single new band appears at ~40 kDa compared to the non-irradiated control. (**b**) MNs containing H2BssUb* with the photoLeu at position 73 of ubiquitin generated the same crosslinked species after irradiation with slightly lower efficiency. Left: SDS-PAGE analysis of

crosslinking reaction. Right ESI-MS analysis of H2BssUb* with the photoLeu incorporated at position 73. This protein was generated according to the semisynthetic route shown in Figure 1b.

a

H2A: 74.62%

MSGRGKQGGK TRAKAKTRSS RAGLQFPVGR VHRLLRKGNY AERVGAGAPV YLAAVLEYLT AEILELAGNA ARDNKKTRII

PRHLQLAVRN DEELNKLLGR VTIAQGGVLP NIQSVLLPKK TESSKSAKSK

Ub: 88.16%

MQIFVKTLTG KTITLEVEPS DTIENVKAKI QDKEGIPPDQ QRLIFAGKQL EDGRTLSDYN IQKESTLHLV LRLRGG

b

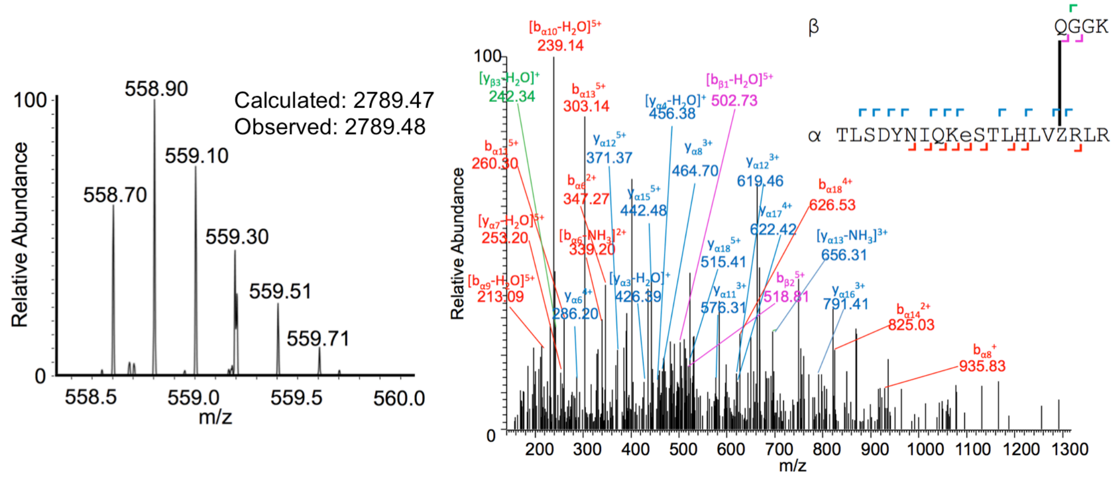

Supplementary Figure 6: MS analysis of crosslinked species within H2BssUb* MNs. MNs containing H2BssUb* were irradiated at room temperature for 25min. The crosslinked mixtures were then separated by non-reducing SDS-PAGE and visualized by GelCode Blue Safe Protein Stain. The crosslinked band was cut from the gel, digested with trypsin and subjected to LC-MS/MS analysis. (**a**) Peptide spectral matches of H2A and Ub. ProteomeDiscoverer (v.1.4, ThermoFisher) and Sequest HT (Thermo Fisher Scientific) search engine nodes were employed to match MS/MS spectra against a database consisting of H2A and Ub, allowing for a parent ion mass window of ±6 ppm, ≤3 missed trypsin cleavages, and methionine oxidation as a variable modification. 74.62% of H2A sequence was covered by identified peptides and 88.16% of Ub

sequence. Peptide identifications at high confidence are highlighted in green, medium confidence in yellow, low confidence in red. (**b**) Raw spectrum of crosslinked peptide, H2A(7-10)-Ub(55-74). The most probable crosslinked peptide, H2A(7-10)-Ub(55-74), was identified by Stavrox[1] (v.3.4.12) and manually inspected using Xcalibur (v. 2.2, ThermoFisher). Left, high-resolution MS spectrum of the parent ion. Right, fragment ion mass spectrum between Ub(55-74) and H2A(1-10). The peptide sequence (with glutamate analogue indicated as 'e' and photo-Leu indicated as 'Z') is annotated to indicate the detected b_n and y_n ions. Ions of the crosslinked α-peptide are represented in red (b_n) and blue (y_n), while ions of the β-peptide are represented in pink (b_n) and green (y_n).

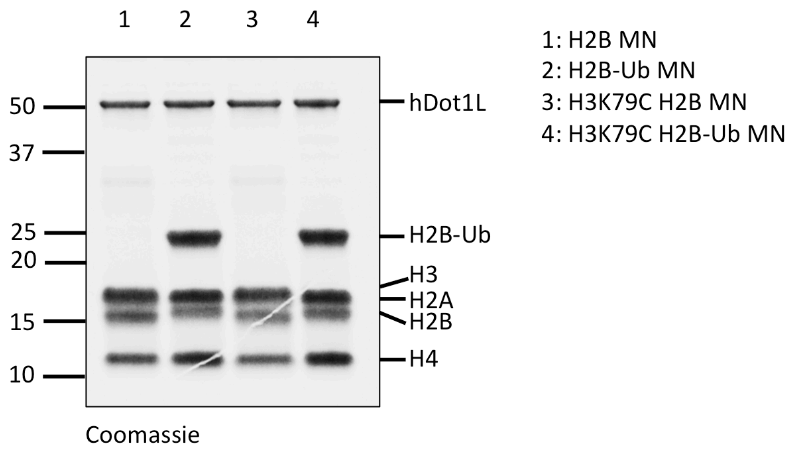

Supplementary Figure 7: hDot1L binds MNs regardless of the presence or absence of H2B-Ub. Biotinylated MNs containing H2B or H2B-Ub, wt H3 or mutant H3K79C were bound to magnetic streptavidin beads respectively, subsequently incubated with 10 equiv. of the catalytic domain of hDot1L for 10min, washed 3 times with wash buffer (25 mM Tris-HCl, 150mM NaCl, pH 7.8), and eluted after incubation with elution buffer (25 mM Tris-HCl, 2M NaCl, pH 7.8). Resulting elutions were separated by non-reducing SDS-PAGE and visualized by GelCode Blue Safe Protein Stain. Lanes correspond to MNs containing: wt H3 and wt H2B (lane 1), wt H3 and H2B-Ub (lane 2), H3K79C and wt H2B (lane 3), H3K79C and H2B-Ub (lane 4).

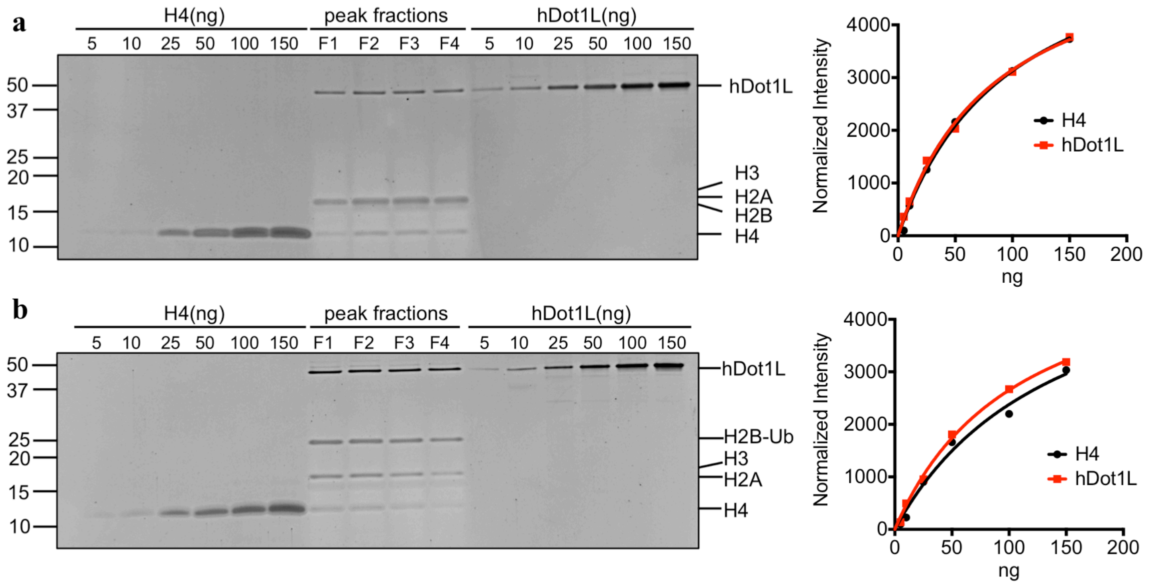

Supplementary Figure 8: Gel quantification of MN-hDot1L complexes using ratiometric standards. H4 standards were used to quantify the amount of MNs in the complex and hDot1L standards were used to quantify the amount of hDot1L in the complex. Plots were fitted into y=Ax/(B+x) to yield standard curves of H4 and hDot1L in each gel. The amount of MNs and hDot1L in each fraction were calculated accordingly. (**a**) SDS-PAGE analysis of the SEC fractions of unmodified MN-hDot1L complex. Average molar ratio of hDot1L/MN was determined as 0.97, around 1. (**b**) SDS-PAGE analysis of the SEC fractions of ubiquitylated MN-hDot1L complex. Average molar ratio of hDot1L/MN was determined as 1.88, around 2.

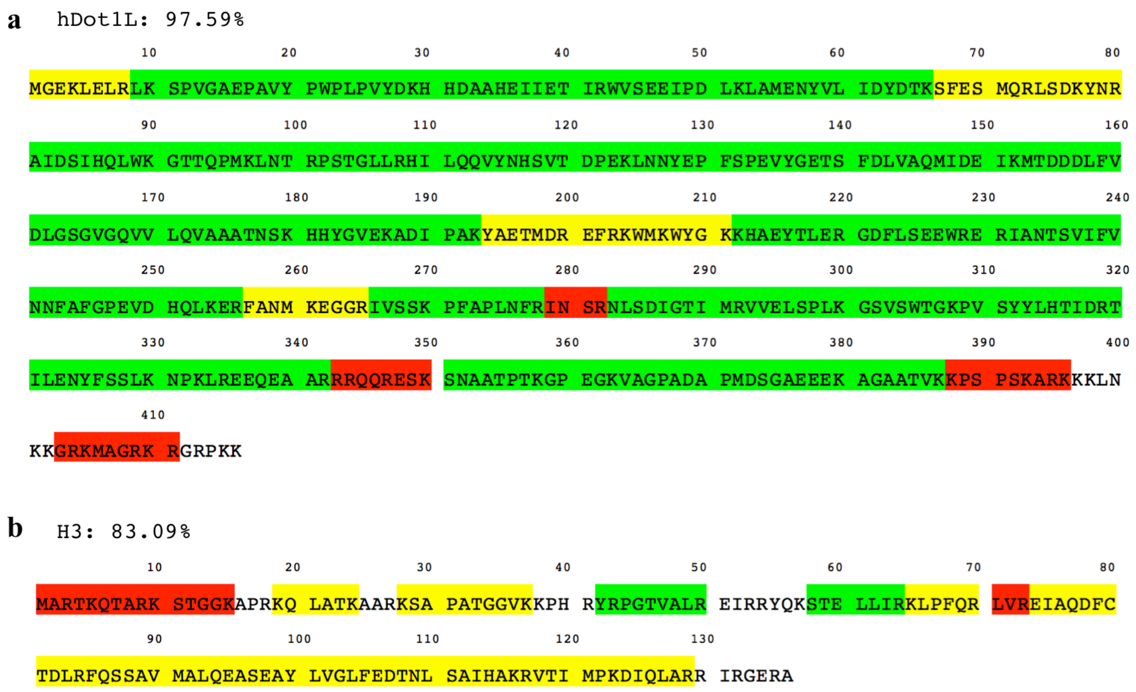

Supplementary Figure 9: MS analysis identifies crosslinked species as H3-hDot1L using MNs containing H3K79[diazirine] and the catalytic domain of hDot1L. ProteomeDiscoverer (v.1.4, ThermoFisher) and Sequest HT (Thermo Fisher Scientific) search engine nodes were employed to match MS/MS spectra against a database consisting of hDot1L and H3, allowing for a parent ion mass window of ±6 ppm, ≤3 missed trypsin cleavages, and methionine oxidation as a variable modification. Peptide identifications at high confidence are highlighted in green, medium confidence in yellow, low confidence in red. (a) Peptide spectral matches of the catalytic domain of hDot1L. 97.59% of hDot1L sequence was covered by identified peptides. (b) Peptide spectral matches of H3. 83.09% of H3 sequence was covered by identified peptides.

```
MSGRGK-GGKG.........H4
MSGRGKQGGK...........H2A
```

Supplementary Figure 10: Alignment of first 10 amino acids of *xenopus* H4 and H2A. Within this region, H2A differs from H4 by only a single residue, the insertion of a glutamine residue.

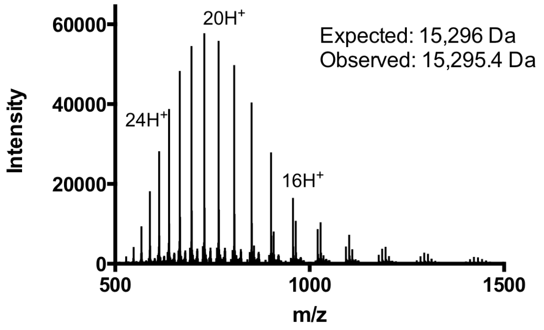

Supplementary Figure 11: Characterization of H3K79C[diazirine] by ESI-MS.

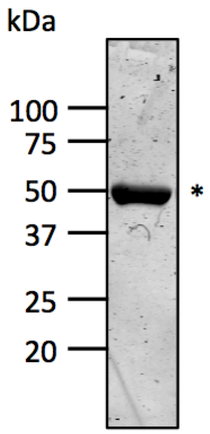

Supplementary Figure 12: Analysis of purified hDot1L. Purified untagged catalytic domain of hDot1L (expected size 47 kDa) was analyzed by 15% SDS-PAGE and Coomassie staining. hDot1L band is marked with an asterisk.

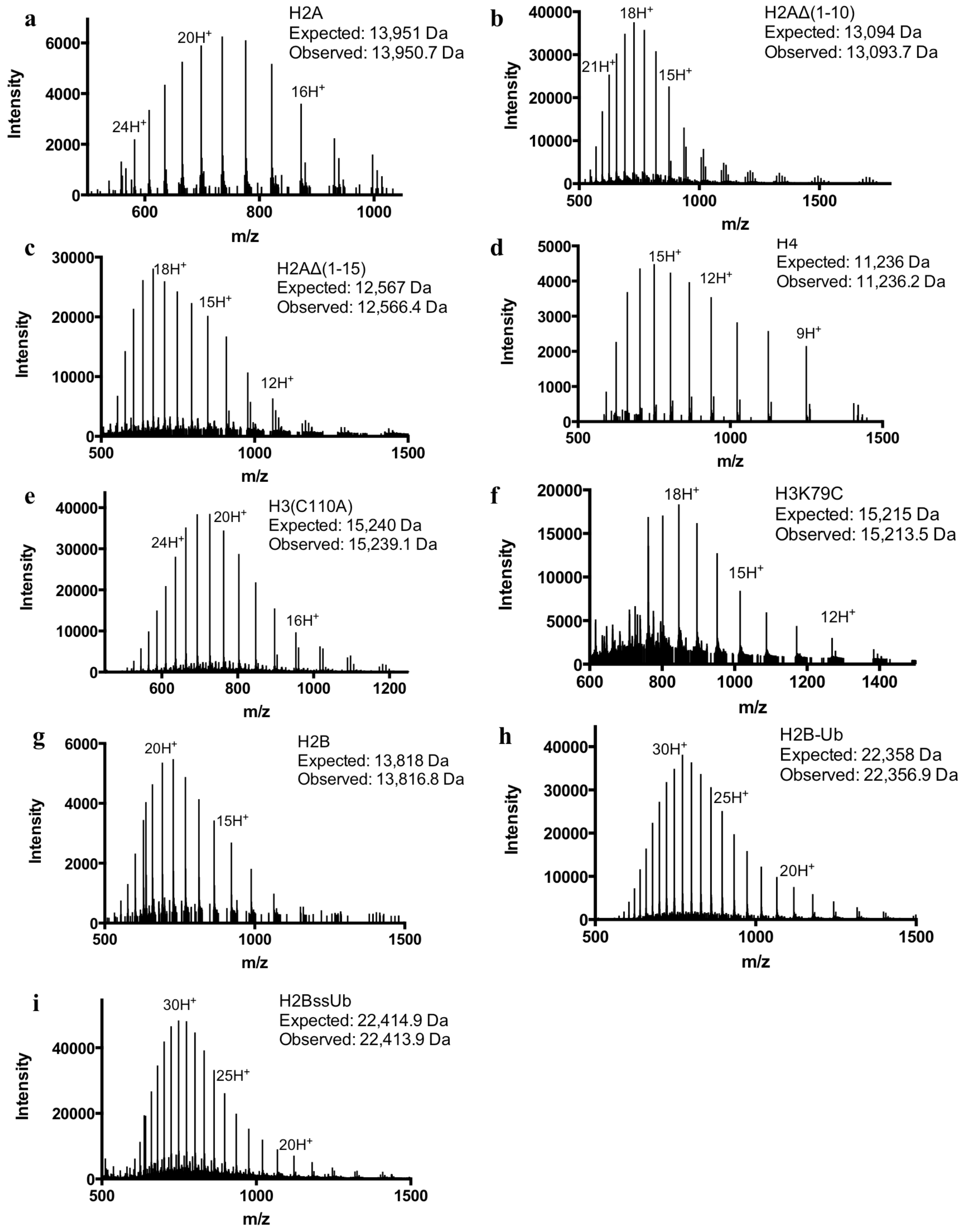

Supplementary Figure 13: Characterization of unmodified and mutant histones. (a) ESI-MS characterization of H2A. (b) ESI-MS characterization of H2AΔ(1-10). (c) ESI-MS

characterization of H2AΔ(1-15). (d) ESI-MS characterization of H4. (e) ESI-MS characterization of H3(C110A). (f) ESI-MS characterization of H3K79C. (g) ESI-MS characterization of H2B. (h) ESI-MS characterization of H2B-Ub. (i) ESI-MS characterization of H2BssUb.

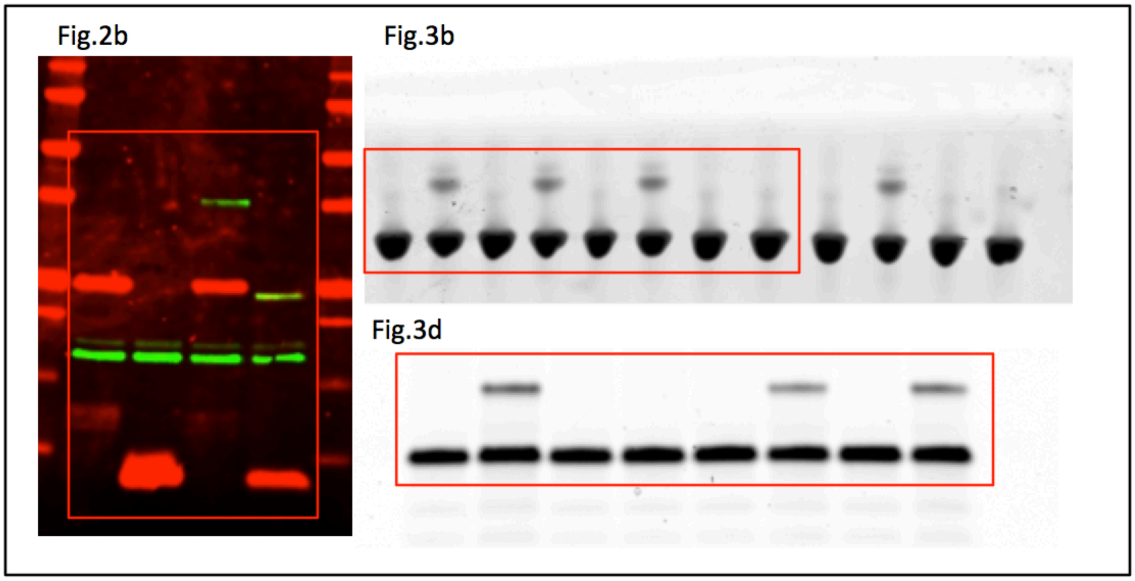

Supplementary Figure 14: Uncropped images of western blots shown in Figure 2b, 3b and 3d. Red boxes show approximate image used for presentation**.**

Supplementary methods

Materials

Dimethylformamide (DMF), dichloromethane (DCM), triisopropylsilane (TIS) were purchased from Sigma-Aldrich (Milwaukee, WI) and used without further purification. Tris(2-carboxyethyl)phosphine hydrochloride (TCEP) was purchased from Thermo Scientific (Rockford, IL). Fmoc amino acids and 2-chloro-trityl chloride(Trityl) resin were purchased from Novabiochem (Darmstadt, Germany) or Bachem (Torrance, CA). Rink AmideChemmatrix was purchased from PCAS Biomatrix (Quebec, Canada). 2-(7-Aza-1H-Benzotriazole-1-yl)-1,1,3,3-tetramethyluronium hexafluorophosphate (HATU) and O-(Benzotriazol-1-yl)-N,N,N',N'-tetramethyluronium hexafluorophosphate (HBTU) were purchased from Genscript (Piscataway, NJ). Trifluoroacetic acid (TFA) was purchased from Halocarbon (North Augusta, SC). The thiol activating reagents 2,2'-dithiobis(5-nitropyridine) (DTNP), 5, 5'-Dithiobis(2-nitrobenzoic acid) (DTNB), and cystamine dihydrochloride were purchased from Sigma-Aldrich Chemical Company (Milwaukee,WI). [^{3}H]-S-adenosyl methionine, were obtained from GE Healthcare (Waukesha, WI). S-Adenosyl-L-homocysteine was obtained from Sigma-Aldrich (Milwaukee, WI).

Chemically competent DH5alpha, BL21(DE3), and BL21(DE3)pLysS cells were

purchased from Novagen (Madison, WI). Restriction enzymes, T4 DNA ligase, chitin resin, and NiNTA resin were obtained from New England BioLabs (Ipswitch, MA). Dynabeads® M-280 Streptavidin were purchased from Life Technologies (Grand Island, NY). Primer synthesis and gene sequencing were performed by Integrated DNA Technologies (Coralville, IA) and Genewiz (South Plainfeld, NJ), respectively. Criterion 15% Tris-HCl, 12% Bis-Tris and 5% TBE gels were purchased from BioRad (Hercules, CA). Centricons were from Sartorius (Goettingen, Germany) and dialysis cassettes were from Pierce (Rockford, IL). PCR purification and gel extraction kits were purchased from Qiagen (Valencia, CA). All other commonly used chemical reagents and solvents were purchased from Sigma-Aldrich (Milwaukee, WI) or Fischer Scientific (Pittsburgh, PA).

Equipment

Analytical RP-HPLC was performed on Hewlett-Packard 1100 and 1200 series instruments equipped with a C18 Vydac column (5 µm, 4.6 x 150 mm) at a flow rate of 1 mL/min. Preparative RP-HPLC was performed on a Waters prep LC system comprised of a Waters 2545 Binary Gradient Module and a Waters 2489 UV detector. Purifications were carried out on a C18 Vydac 218TP1022 column (10 µM; 22 x 250 mm) at a flow rate of 18 mL/min. All runs used 0.1 % TFA (trifluoroacetic acid) in water (solvent A) and 90 % acetonitrile in water with 0.1 % TFA (solvent B). ESI-MS analysis was conducted on a Bruker Daltonics MicrOTOF-Q II mass spectrometer. LC-MS/MS

analyses were performed on a reversed-phase nano-UPLC-MS platform, containing an Easy nLC Ultra 1000 nano-UPLC system that is coupled to an Orbi Elite mass spectrometer (ThermoFisher Scientific) and equipped with a Flex Ion Source (Proxeon Biosystems, Odense, Denmark). Size-exclusion and ion-exchange chromatography were performed on an AKTA FPLC system from GE Healthcare equipped with a P-920 pump and UPC-900 monitor. MALS analysis was performed on a Wyatt MALS system comprised of a DAWN HELEOS II MALS detector and an Optilab T-rEX dRI detector. Scintillation counting was performed on a Perkin Elmer Microbeta2 Liquid scintillation counter.

Mutagenesis of ubiquitin in NPU intein 6XHis tagged vector

Ub(1-63)-NPU-6XHis construct was prepared using the previously described Ub-NPU-6XHis plasmid as a template[2] and mutagenic primers as follows: forward primer (Ub1-63)

5'-CCCTGTCTGACTACAACATCCAGAAATGTTTAAGCTATGAAACG-3' and

reverse (Ub1-63)

5'-CGTTTCATAGCTTAAACATTTCTGGATGTTGTAGTCAGACAGGG-3'.

Mutagenesis of histone H3

H3K79C was prepared using the previously described *xenopus laevis* H3(C110A)

construct in a pET3a plasmid as a template[3] using the mutagenic primers: Forward primer (H3K79C) 5'-GAGAAATCGCCCAAGACTTCTGCACCGATCTTCGCTTCCAGAG-3' and reverse primer (H3K79C) 5'-CTCTGGAAGCGAAGATCGGTGCAGAAGTCTTGGGCGATTTCTC-3'.

Mutagenesis of histone H2A

H2AΔ(1-10) and H2AΔ(1-15) was prepared using the previously described *xenopus laevis* H2A construct in a pET3a plasmid as a template[4] using the mutagenic primers:

H2AΔ(1-10):

Forward primer, 5'-CTTTAAGAAGGAGATATACATATGACCCGCGCTAAGGCC-3'

Reverse primer, 5'-GGCCTTAGCGCGGGTCATATGTATATCTCCTTCTTAAAG-3'

H2AΔ(1-15):

Forward primer, 5'-CCCGAGATGAGCGAGTCTTCATATGTATATCTCCTTCTT-3'

Reverse primer, 5'-AAGAAGGAGATATACATATGAAGACTCGCTCATCTCGGG-3'

Preparation of ubiquitin thioester

All constructs (e.g. Ub(1-63)-NPU-6XHis, Ub(1-75)-NPU-6XHis) were expressed, purified and dialyzed into Buffer B similar to Ub aminoethanethiol constructs and then thiolyzed with mercaptoethane sulfonate (MES) as published.[5] Briefly, ubiquitin and Ub mutants were cleaved from the respective NPU intein fusions, after a dialysis step into

buffer B, by incubation with 10 column volumes of buffer B containing 80 mM MES and 10 mM of Tris(2-carboxyethyl) phosphine, pH 7.2 for 18 h. The eluted proteins, bearing the desired C-terminal MES α-thioester, were subsequently purified by C18 process RP-HPLC employing a gradient of 25-55% B, over 60 min, and the purest fractions were pooled and analyzed by ESI-MS. Typical yields were 10-30 mgs per liter per protein.

Expression of recombinant histones

All histones, including mutant histones (e.g. H3K79C, H2AΔ(1-10), H2AΔ(1-15)), were prepared as previously reported from the indicated expression vectors.[3,4,6] The purest fractions from HPLC purification were pooled and analyzed by ESI-MS (Supplementary Figure 13).

Synthesis of H2BssUb constructs by asymmetric disulfide formation

H2BssUb was prepared according to published protocols.[6] The purest HPLC fractions from HPLC purification were pooled and analyzed by ESI-MS (Supplementary Figure 13).

Generation of H2BssUb conjugate with photoLeu at position 73 of ubiquitin.

This construct was prepared in a similar manner to that of H2B-ubiquitin conjugate **1,** as outlined in Figure 1b. The only difference was use of a synthetic C-terminal ubiquitin

fragment in which photoLeu was incorporated at position 73 to give Ub(64-76)E64C/L73L*. Otherwise, the synthesis was identical to that of **1**.

Preparation of H2B-Ub conjugates by expressed protein ligation

H2B-Ub was prepared using an established sequential expressed protein ligation procedure.[7] Note, the ubiquitin in these semisynthetic constructs harbored a G76A mutation. The purest fractions from HPLC purification were pooled and analyzed by ESI-MS (Supplementary Figure 13).

DNA preparation

Unlabeled and biotinylated 601-147-1 was prepared by PCR as previously described[8] using unlabeled or biotinylated primers, respectively. For large scale mononucleosome formation, a 153bp segment containing the 601 DNA sequence was prepared as previously described[2]. For array formation, a plasmid containing 4 copies of a 177 base pair repeat of the 601 nucleosome positioning sequence (4-177-601) flanked by EcoRV sites was purified from a 6 L culture of DH5alpha cells as previously described.[8]

Supplementary References

1 Götze, M., Pettelkau, J., Schaks, S., Bosse, K., Ihling, C., Krauth, F., Fritzsche, R., Kühn, U. & Sinz, A. StavroX—A software for analyzing crosslinked products in protein Interaction studies. *J. Am. Soc. Mass Spectrom.* **23**, 76-87 (2012).

2 Holt, M. T., David, Y., Pollock, S., Tang, Z., Jeon, J., Kim, J., Roeder, R. G. & Muir, T. W. Identification of a functional hotspot on ubiquitin required for stimulation of methyltransferase activity on chromatin. *Proc. Natl. Acad. Sci. USA* **112**, 10365-10370 (2015).

3 Fierz, B., Chatterjee, C., McGinty, R. K., Bar-Dagan, M., Raleigh, D. P. & Muir, T. W. Histone H2B ubiquitylation disrupts local and higher-order chromatin compaction. *Nat. Chem. Biol.* **7**, 113-119 (2011).

4 McGinty, R. K., Kim, J., Chatterjee, C., Roeder, R. G. & Muir, T. W. Chemically ubiquitylated histone H2B stimulates hDot1L-mediated intranucleosomal methylation. *Nature* **453**, 812-816 (2008).

5 Shah, N. H., Dann, G. P., Vila-Perelló, M., Liu, Z. & Muir, T. W. Ultrafast protein splicing is common among cyanobacterial split inteins: implications for protein engineering. *J. Am. Chem. Soc.* **134**, 11338-11341 (2012).

6 Chatterjee, C., McGinty, R. K., Fierz, B. & Muir, T. W. Disulfide-directed histone ubiquitylation reveals plasticity in hDot1L activation. *Nat. Chem. Biol.* **6**, 267-269

(2010).

7 McGinty, R. K., Köhn, M., Chatterjee, C., Chiang, K. P., Pratt, M. R. & Muir, T. W. Structure–activity analysis of semisynthetic nucleosomes: mechanistic insights into the stimulation of Dot1L by ubiquitylated histone H2B. *ACS Chem. Biol.* **4**, 958-968 (2009).

8 Dyer, P. N., Edayathumangalam, R. S., White, C. L., Bao, Y., Chakravarthy, S., Muthurajan, U. M. & Luger, K. Reconstitution of nucleosome core particles from recombinant histones and DNA. *Methods Enzymol.* **375**, 23-44 (2004).
